# Multivalency as a chemical organization and action principle

**DOI:** 10.3762/bjoc.11.94

**Published:** 2015-05-19

**Authors:** Rainer Haag

**Affiliations:** 1 Institute for Chemistry and Biochemistry, Freie Universität Berlin, Takustr. 3, 14195 Berlin, Germany

**Keywords:** glycoarchitectures, supramolecular chemistry, multivalency, multivalent protein inhibitors, pathogen binding

Multivalency is a key principle in nature to establish strong, but also reversible chemical interactions between two units, e.g., a receptor and a ligand, viruses and host cells, or between two cell surfaces. Multivalent binding is based on multiple simultaneous molecular recognition processes and plays an important role in the self-organization of matter, in biological recognition processes as well as in signal transduction in biological systems. The targeted development of multivalent molecules is not only used for the strong inhibition of proteins and prevention of pathogen infections, but also allows for the selective production of functional molecular architectures and surface structures as well as the controlled interaction of multivalent surfaces. The chemical and biological mechanisms and the influence of scaffold architectures with different dimensions for multivalent interactions have not been comprehensively explored. Thus, the experimental and theoretical understanding of defined oligovalent binding systems requires further detailed understanding in the gas phase, in solution and on surfaces.

**Figure 1:**
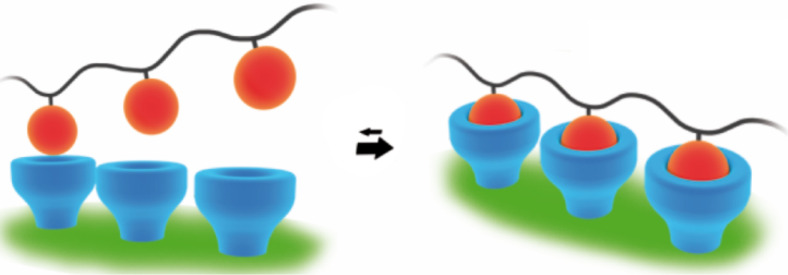
Multivalent interactions shift the equilibrium and enhance the binding strength. Reprinted with permission from [1]. Copyright 2012 Wiley-VCH.

Frequently the interaction of a single ligand with an acceptor (monovalent interaction) in many cases can be quantitatively understood. This, however, is completely different, when several covalently linked ligands of the same or of a similar nature bind to an ensemble of acceptors (multivalent interactions) [1]. Due to the multiple additive (in some cases cooperative) interactions the equilibrium will shift and bond reinforcement occurs (Figure 1). Also, the kinetically controlled dissociation can become very slow to almost non-existent. Multivalency is also dependent on the size, shape and flexibility of the scaffold architecture, especially for the interfacial interaction with biological systems.

In order to obtain a deeper understanding of multivalent interactions this Thematic Series in the *Beilstein Journal of Organic Chemistry* puts its focus to unravel new cellular interactions that are highly relevant [2] and systematically combines theoretical exploration of defined multivalent supramolecular interactions [3] as well as new supramolecular designer systems [4–5]. The influence of spacer length and flexibility on the binding affinity of ligands [6] will be examined as well as the mechanical stability of complexes [7]. Furthermore, the Thematic Series covers the synthesis of various new glycoarchitectures for multivalent interactions [8–12] and studies the scope of multivalent lectin-glycointeractions in galectins [13], with iminosugars [14] and carbohydrate mimetics [15]. This Thematic Series in the *Beilstein Journal of Organic Chemistry* also investigates the enhanced multivalent binding of protein scaffolds [16], peptide–polymer interactions [17–20] tripodal-catecholates [21] and polycatechol–surface interactions [22] as well as multivalent organocatalyts [23]. Finally, multivalent dendritic poly(arginine/histidine)-siRNA complexes are evaluated regarding their transfection efficiency [24].

In the future a deeper understanding of multivalent interactions at all length scales from the nanometer to the micrometer range is crucial for solving important problems and for the development of new systems in the fields of life and materials science. In order to address this highly complex and long-term challenge, the interdisciplinary cooperation of scientists with different expertise ranging from biochemistry to theory is essential.

Rainer Haag

Berlin, May 2015
